# Effects of Concomitant Antibiotics Use on Immune Checkpoint Inhibitor Efficacy in Cancer Patients

**DOI:** 10.3389/fonc.2022.823705

**Published:** 2022-02-09

**Authors:** Shuai Jiang, Shuai Geng, Qian Chen, Chen Zhang, Mengfei Cheng, Yang Yu, Shuo Zhang, Ning Shi, Mei Dong

**Affiliations:** ^1^ Department of Pharmacy, Harbin Medical University Cancer Hospital, Harbin, China; ^2^ Department of Pharmacy, Strategic Support Force Medical Center, Beijing, China; ^3^ Department of Pharmacy, Beijing Boren Hospital, Beijing, China; ^4^ Department of Medical Imaging, Strategic Support Force Medical Center, Beijing, China

**Keywords:** cancer, immune checkpoint inhibitors (ICIs), antibiotics, progression-free survival (PFS), overall survival (OS)

## Abstract

**Objective:**

Immune checkpoint inhibitors (ICIs) have changed the outcomes of a variety of cancers in an unprecedented manner. Gut microbiome plays a crucial regulatory role in the antineoplastic therapy of ICIs, which can be influenced by antibiotic (ABX) administration. In this efficacy evaluation, we aimed to clarify the correlations of ABX administration with the survival of cancer patients receiving ICIs treatment.

**Method:**

The eligible literatures were searched using PubMed, Cochrane Library, Web of Science, and Clinical trials.gov databases before Nov 2021. The correlations of ABX administration with progression-free survival (PFS) and overall survival (OS) were determined using Hazard ratios (HRs) coupled with 95% confidence intervals (CIs).

**Results:**

A total of 12 studies enrolling 6010 cancer patients receiving ICIs treatment were included in this efficacy evaluation. ABX administration was significantly correlated worse PFS (HR=1.60, 95%CI=1.33-1.92, P<0.00001) and OS (HR=1.46, 95%CI=1.32-1.61, P<0.00001). Similar results were found in the subgroup analysis of non-small cell lung cancer (NSCLC), renal cell carcinoma (RCC) and melanoma.

**Conclusions:**

ABX use during ICIs treatment of cancer may significantly shorten PFS and OS. ABX should be used cautiously in cancer patients receiving ICIs. However, further validations are still essential due to existing publication bias.

## Introduction

In recent years, as new antitumor drugs, Immune checkpoint inhibitors (ICIs) have significantly improved the prognosis of patients with various types of tumor which brings a “Immune Era” with representative drugs included programmed cell death 1(PD-1)/programmed cell death ligand 1(PD-L1) inhibitors and cytotoxic T lymphocyte-associated antigen 4(CTLA4) antibodies (1). Gut microbes play an important role in regulating the efficacy and toxicity of cancer immunotherapy (2, 3). Phase I clinical trials in animal models suggested that gut microbes may be key modulators of ICIs efficacy and toxicity. Routy et al. (4) confirmed that transplanting intestinal microorganisms from patients into sterile mice could enhance the anti-tumor efficacy of PD-1 inhibitors. Therefore, it is suggested that the response of cancer patients to ICIs may be influenced by conditions of altering the composition of gut microbes, including dysbiosis due to antibiotic use (ABX).

The relationship between ABX use and cancer therapy has been extensively studied, especially in the prevention of perioperative infection and immunosuppressive associated infection induced by chemoradiotherapy (5). There are few reports on the role of ABX in the treatment of ICIs in tumor patients, but the conclusions varied greatly which were influenced by the type and duration of administration. Several studies have compared the effects of ABX on clinical outcomes before/during/after the use of ABX with those without, and some patients have negative effects on treatment response and survival, such as Huang (6), Lurienne (7), etc. Other studies (8, 9) have shown no significant correlation between ABX administration during or before ICIs treatment and remission rates and PFS in cancer patients. Therefore, the prognostic effect of ABX in the treatment of ICIs is still unclear, and the comprehensive and objective evaluation is urgently needed. In the present study, we evaluated the efficacy of 12 studies in 6010 patients treated with ICIs and analyzed the association between ABX use and survival, with the expectation that the results would contribute to the individualized clinical management of cancer immunotherapy and the improvement of patient survival, we evaluated 12 studies of 6010 patients treated with ICIs and analyzed the association between ABX use and survival, with the aim of improving individual clinical management and patient survival during cancer immunotherapy.

## Methods and Materials

We followed the Preferred Reporting Items for Systematic Reviews and Meta-Analyses (PRISMA) guidelines to report our meta-analysis. We systematically searched domestic and foreign literatures on antibiotic application versus non-antibiotic application before, during or after ICIs treatment in cancer, and systematically evaluated the impact of antibiotics in cancer treatment on the efficacy of ICIs.

### Search Strategy

We use a variety of retrieval tools to conduct a comprehensive literature search. (1) Computer literature database search: ①Chinese search terms included “immune checkpoint inhibitors”, “cancer”, “immunotherapy”, “programmed cell death protein 1”, “programmed cell death protein ligand 1”, “cytotoxic T lymphocyte antigen 4”, etc. ②English keywords included “ICIs”, “cancer”, “immunotherapy”, “PD-1”, “PD-L1”, “CTLA-4”, etc. ③Different combinations of PubMed, Cochrane Library, Embase and EBSCO evidence-based medicine databases were searched, including title, abstract and keywords, and the search period was from self-establishment to November 2021.

### Study Selection

As immunotherapy becomes more widely used in many cancer patients, some studies showed that both PFS and OS were significantly reduced in patients treated with ICIs and antibiotics. Therefore, it is important to determine whether antibiotics affect the prognosis of patients treated with ICIs. At present, systematic evaluation in this field mainly focuses on multi-factor analysis, while antibiotic single-factor analysis is rare. In order to further systematically evaluate the single factor effect of antibiotic and ICIs combination, the following inclusion criteria were used: (1) Included population: solid tumor patients treated with ICIs; (2) Literature type: prospective or retrospective study; (3) Interventions: antibiotic use before, during, or after ICIs treatment versus no antibiotic use; (4) Outcome measures: PFS and/or OS-related hazard ratios (HRs) with 95% confidence intervals (95%CI). Meanwhile, the following exclusion criteria were used: (1) No control group was established; (2) Repeatability study; (3) Non-Chinese and English literature; (4) HRs literature for PFS and/or OS is not provided

### Data Extraction and Quality Assessment

Data were extracted from the eligible studies included according to the PRISMA statement: author’s name, year of publication, type of publication (such as publication poster and abstract), country patient sample size, HRs and 95%CI of antibiotic treatment window, PFS was defined as spanning from randomization to either recurrence or death, and OS was defined as spanning from randomization to death. The Newcastle-Ottawa scale (NOS) was used to evaluate the quality of the literature (10), and the quality of the included studies was evaluated according to the following 8 criteria: (1) the representativeness of the exposure cohort; (2) the non-exposure cohort Selection; (3) Determination of exposure method; (4) No subject had an outcome event before the start of the study; (5) Comparability of exposure cohort and non-exposure cohort; (6) Evaluation of outcome events; (7) Whether the follow-up time is long enough; (8) Whether the follow-up is complete. Documents rated 7-9 points are considered “high” quality, 4-6 points are “fair”, and 3 points or lower are considered “low”. The quality evaluation is carried out independently by two researchers and cross-checked. If there is a disagreement, the third researcher is requested to assist in the resolution.

### Statistical Analysis

Meta-analysis was performed using RevMan 5.2 software provided by the Cochrane Collaboration. All the HRs included in the study were pooled together to provide an overall effect size. Cochrane χ^2^ test was used to analyze the heterogeneity between studies, and I^2^ was used to evaluate the heterogeneity. When P > 0.1 and I^2^ < 50%, there was no statistical heterogeneity for RCTs, and the fixed-effect model was used. On the contrary, the random effect model was adopted on the premise of excluding clinical heterogeneity. An inverted funnel plot was used to analyze publication bias, and sensitivity analysis was conducted for each included literature. The experimental bias of included literature was also discussed.

## Results

### Search Results and Patient Characteristics

**Figure 1:**
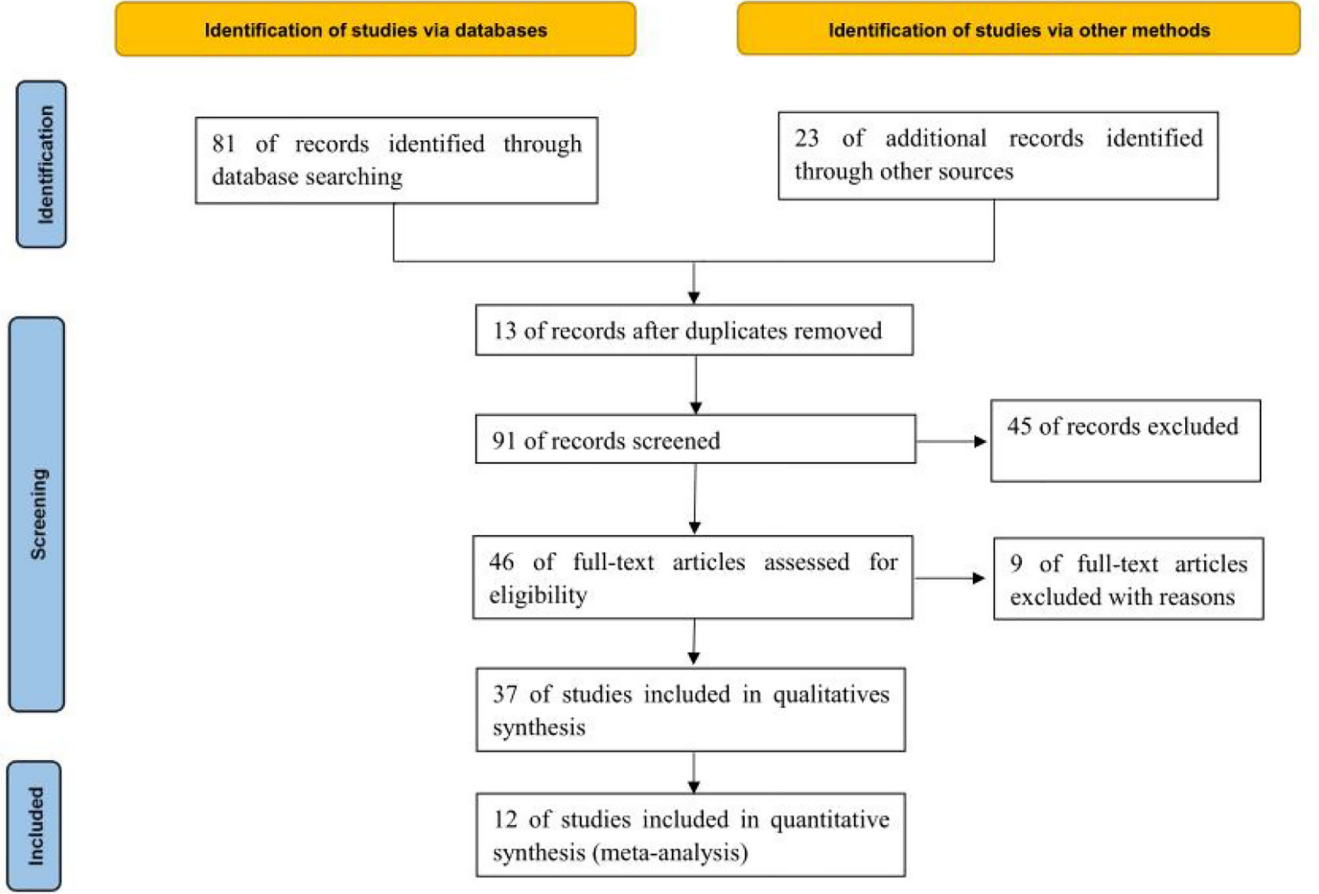
PRISMA Flow chart of article selection.

Through database retrieval, 81 relevant literatures were obtained, including 8 Chinese literatures, 73 English literatures, 23 conference papers and abstracts, and 67 duplicated literatures, case reports, reviews and irrelevant contents were excluded. 37 literatures were screened strictly in accordance with the above screening process, and finally 12 (11–22) studies were included in the quantitative analysis. A total of 6010 cancer patients were involved, of whom 1414 were treated with ABX in the treatment window of ICIs, as shown in **Figure 1**.

A total of 6010 cancer patients meeting the requirements were included in the 12 literatures, including 1414 patients who received antibiotics during ICIs treatment and 4596 patients who did not receive antibiotics. All 12 literatures were of high quality, as shown in **Table 1**.

**Table 1:** Basic characteristics of included studies.

First Author	Year	Journal	Country	Type of Study	Type of Cancer	Patients (ATB+/ATB-)	mPFS,ABX+ vs ABX- (months)	mOS,ABX+ vs ABX-(months)	HR for PFS [95% CI]	p-Value for PFS	HR for OS [95% CI]	p-Value for OS	Quality
Umang Swami (11)	2020	Antibiotics	USA	Retrospective	Melanoma	30/169	NA	NA	1.28 [0.80,2.04]	0.30	1.73 [1.00,2.99]	0.05	7
Cortellini (12)	2021	Annals of oncololgy	UK	Retrospective	NSCLC	47/302	5.6 vs 6.3	11.2 vs 16.6	1.25 [0.84,1.84]	0.26	1.63 [0.99,2.68]	0.05	7
KAZUYUKI HAMADA (13)	2021	Anticancer Research	Japan	Retrospective	NSCLC	18/69	6.4 vs 19.9	20.6 vs 72.8	3.16 [1.55,6.25]	0.002	1.99 [0.91,4.09]	0.082	7
KOSUKE UEDA (14)	2019	Anticancer Research	Japan	Retrospective	RCC	5/31	2.8 vs 18.4	NA	6.52 [1.86,21.42]	0.0004	NA	NA	7
Anne Schett (15)	2019	Cancer Chemotherapy and Pharmacology	Switzerland	Retrospective	NSCLC	33/218	1.4 vs 5.5	1.8 vs 15.4	1.27 [0.94,1.71]	0.12	1.74 [1.24,2.44]	0.001	7
Lalani-1 (16)	2019	European Urology Oncology	Canada	Retrospective	RCC	31/146	NA	NA	1.96 [1.20,3.20]	0.007	1.44 [0.75,2.77]	0.27	7
Lalani-2 (16)	2019	European Urology Oncology	Canada	Retrospective	RCC	709/3435	NA	NA	1.16 [1.04,1.30]	0.008	1.25 [1.10,1.41]	0.001	
Chirayu Mohindroo (17)	2020	Cancer Medicine	USA	Retrospective	PDAC	209/580	4.4 vs 2.0	13.3 vs 9.0	2.08 [1.44,3.01]	0.0001	2.08 [1.44,3.01]	0.0001	7
Arielle Elkrief (18)	2019	OncoImmunology	Canada	Retrospective	Melanoma	10/74	2.4 vs 7.3	10.7 vs 18.3	3.57 [1.36,9.40]	0.01	1.92 [0.76,4.87]	0.17	7
L. Derosa-1 (19)	2018	Annals of oncololgy	France	Retrospective	NSCLC	48/239	1.9 vs 3.8	7.9 vs 24.6	1.5 [1.0,2.2]	0.03	4.4 [2.6,7.7]	0.01	7
L. Derosa-2 (19)	2018	Annals of oncololgy	France	Retrospective	RCC	16/121	1.9 vs 7.4	17.3 vs 30.6	3.1 [1.4,6.9]	0.01	3.5 [1.1,10.8]	0.03	
Laura M. Chambers (20)	2021	Gynecologic Oncology	USA	Retrospective	GC	58/101	7.3 vs 6.8	11.6 vs 19.5	0.96 [0.59,1.54]	0.85	1.20 [0.70,2.09]	0.51	7
Nadina Tinsley (21)	2020	The Oncologist	UK	Retrospective	NSCLC, others	92/291	3.1 vs 6.3	10.4 vs 21.7	1.401 [1.028,1.920]	0.033	1.4723 [1.038,2.107]	0.033	7
Hyunho Kim (22)	2019	BMC Cancer	Korea	Retrospective	NSCLC, others	108/234	2.0 vs 4.0	5.0 vs 17.0	1.715 [1.264,2.326]	0.001	1.785 [1.265,2.519]	0.001	7

NSCLC, Non-small cell lung cancer; RCC, Renal cell carcinoma; PDAC, Pancreatic ductal adenocarcinoma; GC, Gynecological cancer; PFS, Progression free survival; OS,Overall survival; ABX, Antibiotics; ABX+, Antibiotics exposure; ABX-, No antibiotics exposure; HR, Hazard ratio; NA, Not available.

### Meta-Analysis Results

#### Effect of Concomitant ABX Use on PFS of ICIs

**Figure 2:**
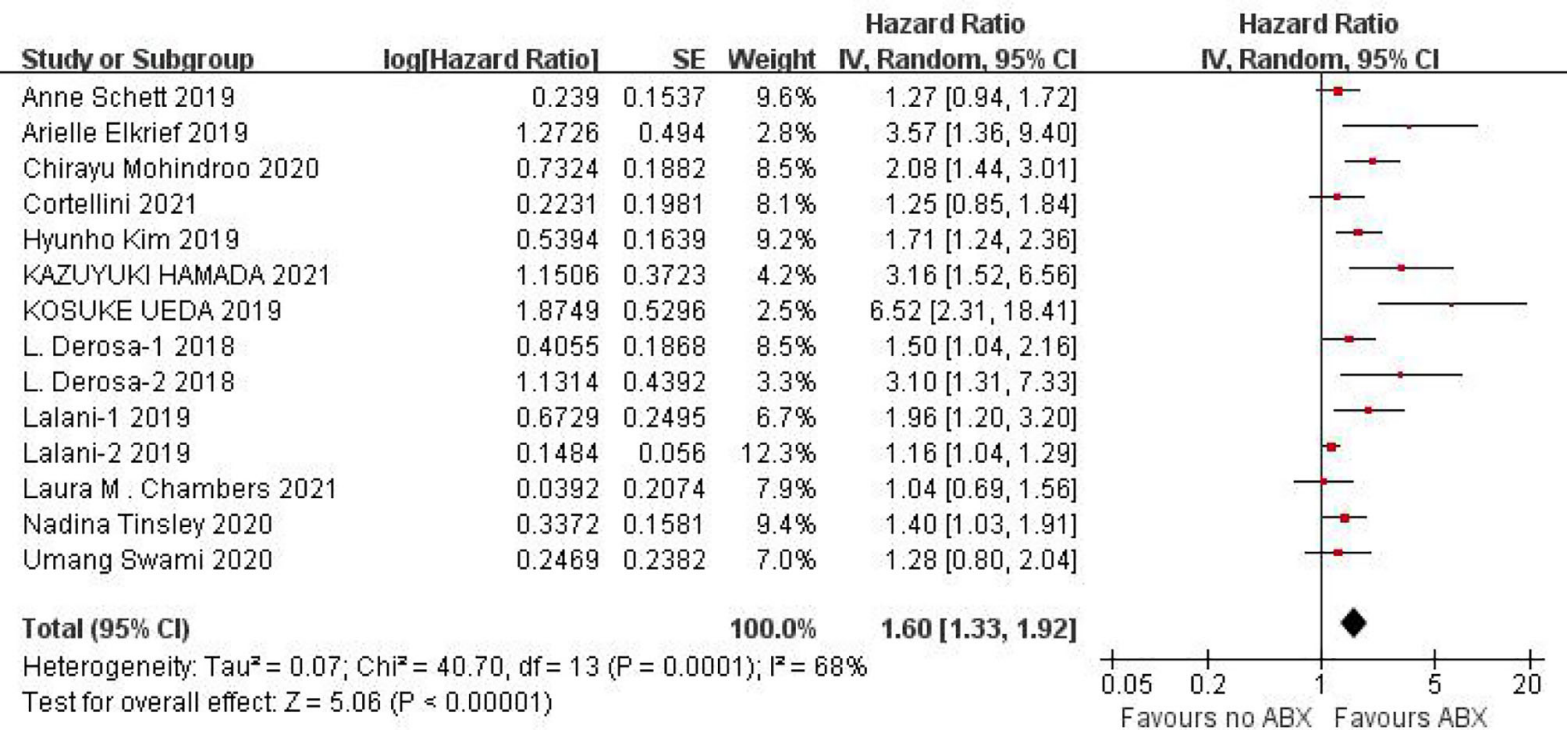
Meta-analysis results of PFS between antibiotics exposed group and non-exposed group.

**Figure 3:**
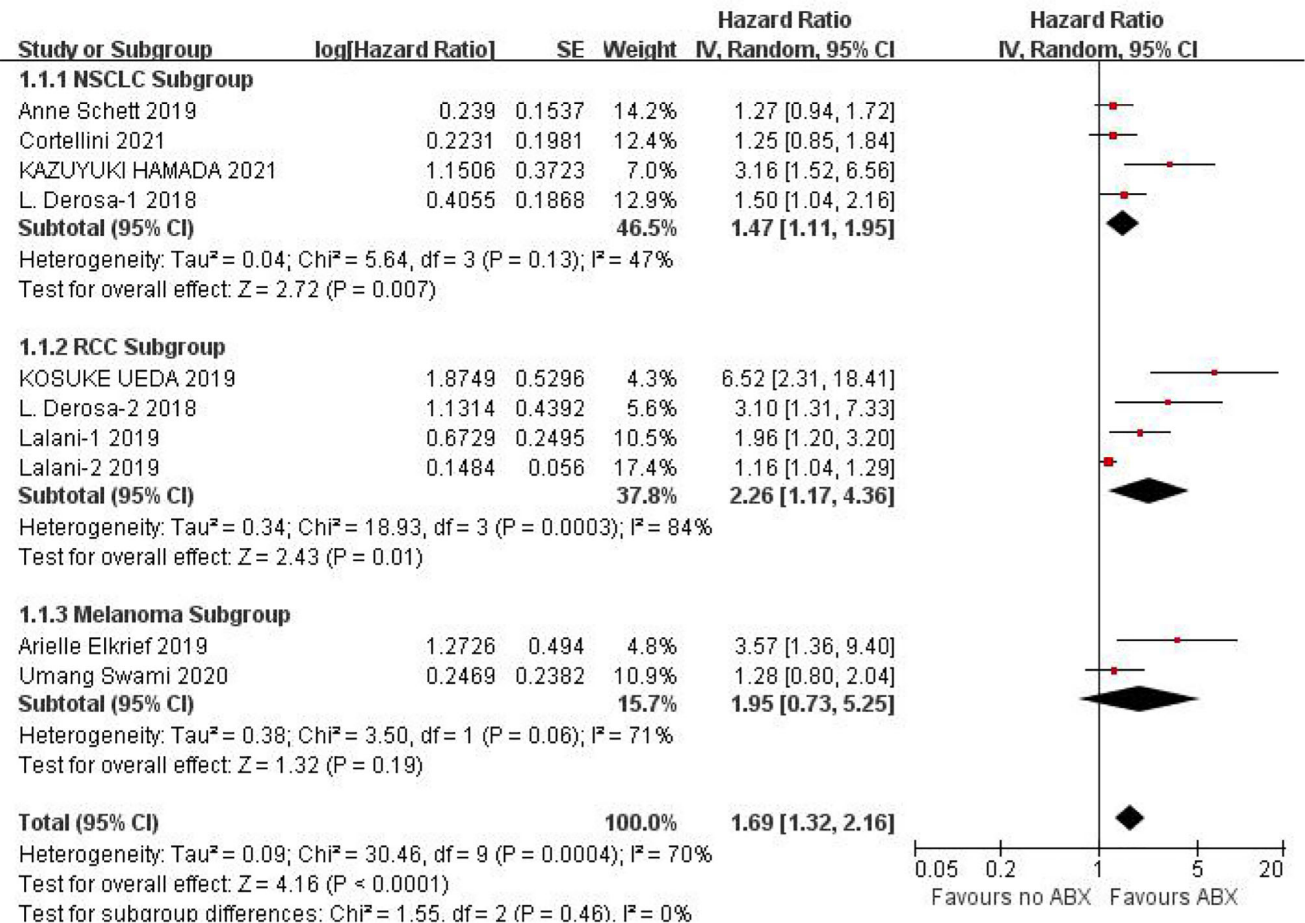
Meta-analysis results of PFS subgroups between antibiotics exposed group and non-exposed group.

PFS data could be obtained from 12 studies for heterogeneity analysis, I^2^ = 68%,P=0.0001. There was statistical heterogeneity among studies, and random effect model was used for analysis. As shown in **Figure 2**, HR=1.60 (95%CI=1.33-1.92, P<0.00001) these results suggest that the use of antibiotics in the cancer immunotherapy window can significantly shorten PFS. In view of the heterogeneity, it was analyzed that the cause might be caused by different cancer diseases. Furthermore, subgroup analysis of PFS based on different cancers (NSCLC, RCC and Melanoma) showed that there was no heterogeneity among studies in the NSCLC group (I^2^ = 47%, P=0.13) and small heterogeneity among studies in the RCC group (I^2^ = 84%, P=0.0003). There was no heterogeneity among studies in the Melanoma group (I^2^ = 71%, P=0.06), as shown in **Figure 3**.

#### Effect of Concomitant ABX Use on OS of ICIs

**Figure 4:**
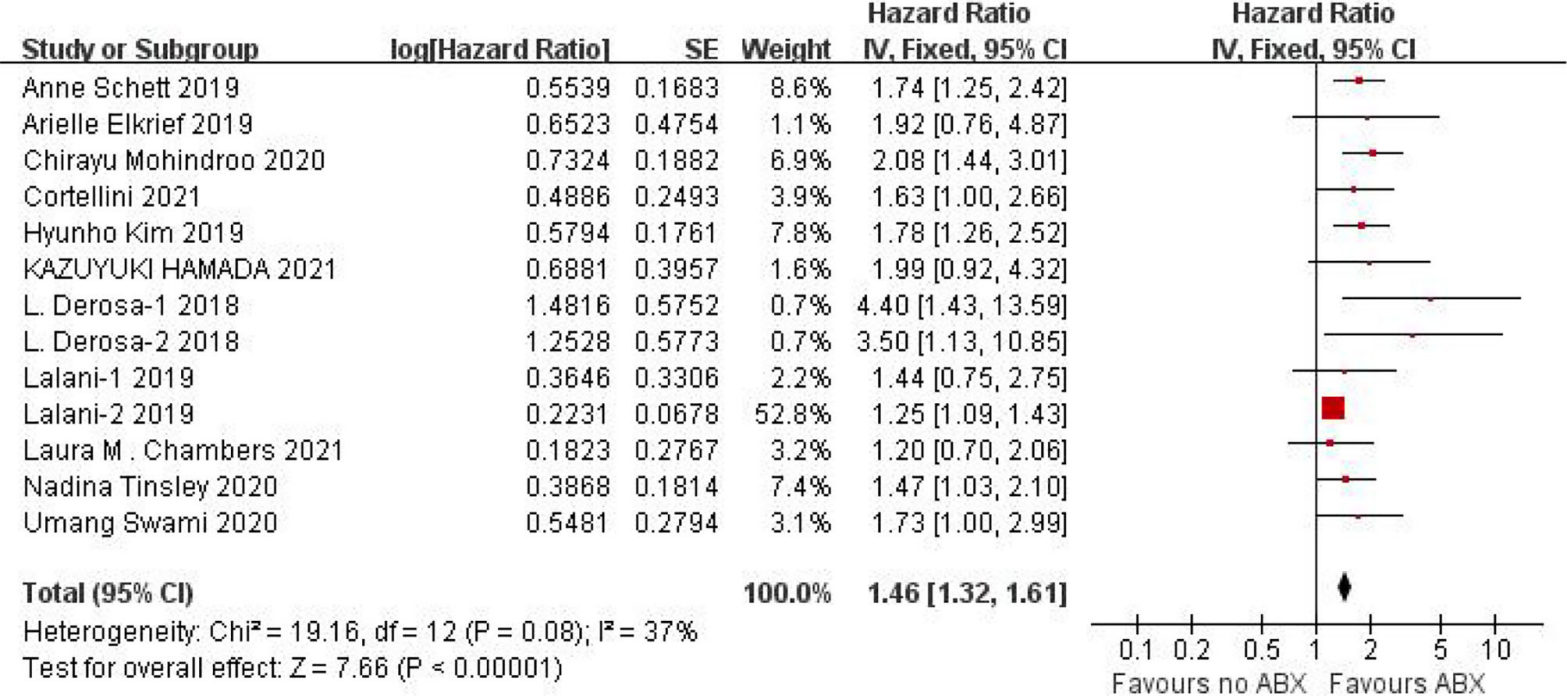
Meta-analysis results of OS between antibiotics exposed group and non-exposed group.

We obtained OS data from 11 studies and conducted heterogeneity analysis(I^2^ = 37%, P=0.08). There was no statistical heterogeneity between studies and fixed effect model was used for analysis. The results showed that HR=1.46 (95%CI=1.32-1.61, P < 0.00001), suggesting that the application of antibiotics in the immunotherapy window of cancer patients can significantly shorten OS, as shown in **Figure 4**.

### Sensitivity Analysis

The pooled HRs for PFS were not significantly different after excluding one study at a time in the sensitivity analysis, ranging from 1.52 [95% CI=1.29-1.80, after excluding KOSUKE’s study (14)] to 1.67 (95%CI=1.37-2.02, after excluding Laura M.Chambers’s study (20)). Moreover, the pooled HRs for OS also did not significantly change in the sensitivity analysis. The overall HRs ranged from 1.42 [95%CI=1.29-1.57, after omitting Chirayu Mohindroo’s study (17)] to 1.47 [95%CI=1.33-1.62, omitting Laura M. Chambers’s study (20)].

### Publication Bias

**Figure 5:**
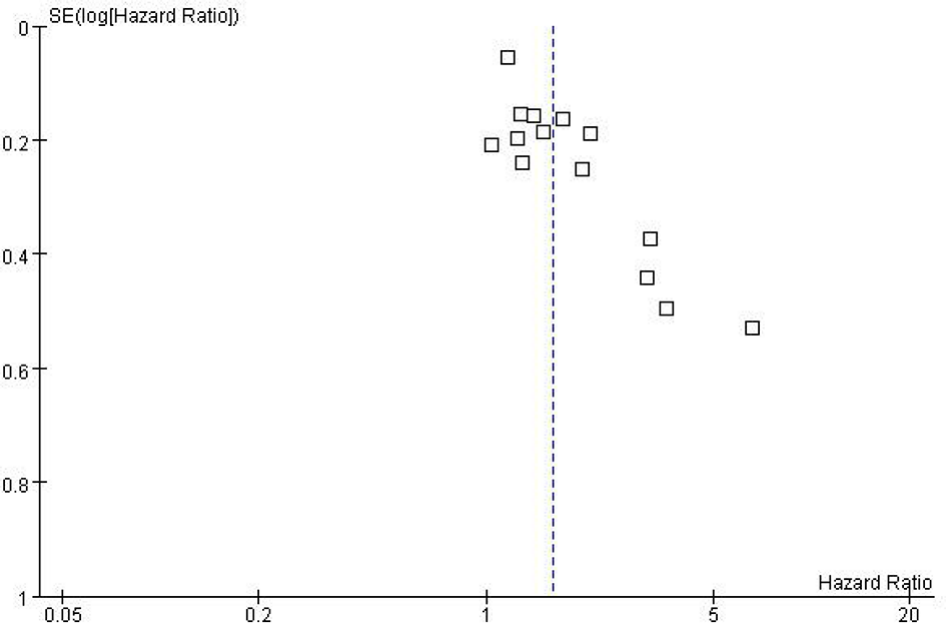
Funnel Plot of PFS.

**Figure 6:**
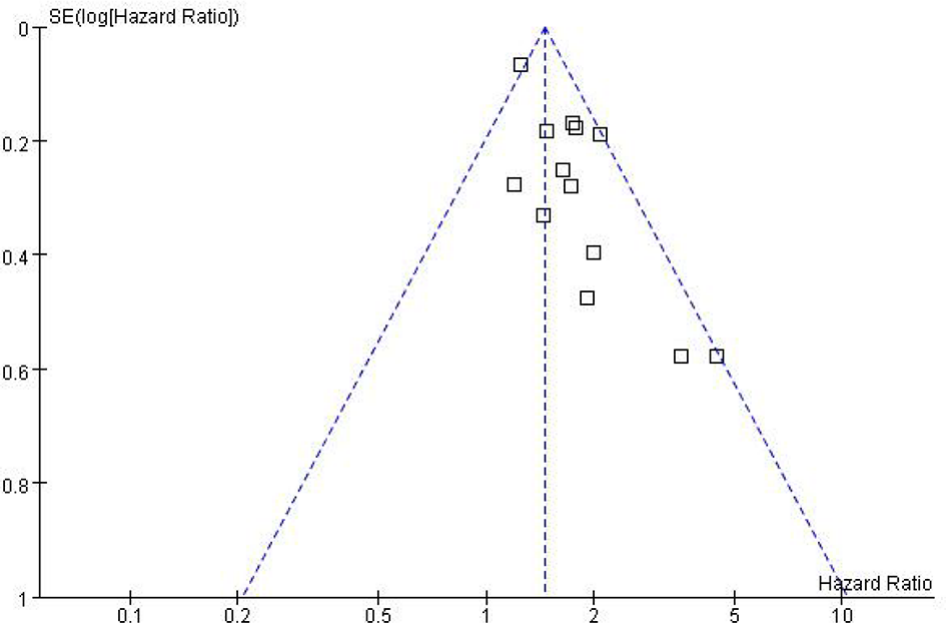
Funnel Plot of OS.

While performing Meta-analysis and comparison of PFS and OS data indicators, an inverted funnel plot was drawn for the included studies. The results showed that PFS has publication bias. Analysis of the reasons may be caused by different types of cancer. Therefore, it is necessary to conduct subgroup analysis and discussion. The OS funnel plot was symmetrical and mainly concentrated in the middle and upper part. Only a few studies may be less rigorous in design, poor research methods and other factors lead to the outside of the inverted funnel chart, suggesting a small bias, as shown in **Figures 5** and **6**.

## Discussion

Immunotherapy has now become one of the important and effective treatment methods for various cancers. In the first-line anti-tumor treatment, KEYNOTE024 (23) and KEYNOTE042 (24) clinical studies have shown that pembrolizumab single-agent contrast chemotherapy can significantly prolong the PFS and OS of PD-L1 (TPS≥50%) NSCLC patients; Keynote-021 (25), Keynote-189 (26), Keynote-407 (27) found that pembrolizumab combined with chemotherapy compared with chemotherapy can significantly prolong the PFS and OS of patients. With the advent of different types of immune checkpoint inhibitors and their gradual introduction into health insurance coverage, the total cost of immunotherapy for cancer patients has gradually decreased, thus enabling an increasing number of cancer patients to benefit from immunotherapy (28, 29). In the era of precision treatment, it is necessary to continue finding ways to further improve the clinical efficacy of immune checkpoint inhibitors.

In recent years, researchers have gradually realized that gut microbes may be a key factor in improving the prognosis of cancer patients (30–32). A lot of evidence shows that the application of ABX is related to the clinical efficacy of cancer immunotherapy. Gajewski et al. (33) found that bifidobacteria enhanced the anti-tumor effect of PD-L1 inhibitors in experimental mice models. In 2018, the team analyzed the composition of the fecal flora of 42 patients with metastatic melanoma, further revealing that the composition of the intestinal flora is significantly related to the effectiveness of PD-1 inhibitor immunotherapy (34). The influence of gut microbes on the efficacy of ICIs has become a research hotspot. However, in patients treated with ICIs, the predictive role of ABX exposure remains unclear. In this study, we evaluated the impact of ABX on the survival of cancer patients treated with ICIs based on multiple tumor types (including NSCLC, melanoma, RCC, etc.) and different dimensions. The results showed that the combined use of ABX is associated with the shortened PFS and OS, and ABX may be a negative prognostic factor for malignant tumors treated with ICIs.

The influence mechanism of ABX on ICIs response is as follows: First of all, the inherent anti-inflammatory effects of ABX, such as quinolone drugs can reduce the levels of pro-inflammatory cytokines (such as interleukin-1, tumor necrosis factor-α) and macrolide drugs, reduce T cell responses, and thereby ICIs have a potential antagonistic effect (35). Secondly, the modification of the intestinal microbiota by ABX will lead to the selection of bacterial species, which will have a negative impact on the response of ICIs. In animals, the transplantation of certain “favorable” bacteria can restore the response to ICIs after broad-spectrum antibiotic treatment (23). Third, the use of ABX affects the diversity of intestinal microbes, which is related to the negative reaction of anti-PD-1 immunotherapy (36). Finally, some ABX independent of ICIs may also have an inherent negative effect on the clinical course of malignant tumors by promoting canceration and metastasis (37).

Due to the poor physical condition and low immunity of cancer patients, the incidence of infection is relatively high, and the probability of using antibiotics is relatively high. Due to the poor physical condition and low immunity of cancer patients, the incidence of infection and the use of antibiotics are relatively higher. This study shows that the application of antibiotics during ICIs treatment of cancer can shorten the PFS (HR=1.60, 95%CI=1.33-1.92, P<0.00001) and OS (HR=1.46, 95%CI=1.32-1.61, P<0.00001) of cancer patients, the results are significantly different. In view of the small heterogeneity of PFS, we analyzed that its source may be related to different cancer types, so we conducted subgroup analysis according to cancer types. The results of subgroup analysis showed that NSCLC (HR=1.47, 95%CI=1.11-1.95, P=0.007), RCC (HR=2.26, 95%CI=1.17-4.36, P=0.01), melanoma (HR=1.95, 95%CI=0.73-5.25, P=0.19). There is no heterogeneity among the studies in the NSCLC group (I^2^ = 47%, P=0.13), there is little heterogeneity among the studies in the RCC group (I^2^ = 84%, P=0.0003), and there is no heterogeneity among the studies in the Melanoma group Heterogeneity (I^2^ = 71%, P=0.06).

However, this study also has some limitations. First, our research is essentially based on a meta-analysis of available data from published literature. Although we have made a lot of efforts to collect as much information as possible, many important details of the included studies, such as heterogeneous populations, tumor types, and patient characteristics have limited our further analysis to a certain extent and affected our results. In addition, due to the rare sequencing evidence, we have not been able to discuss the microbiome changes of patients receiving ABX before and/or during ICI treatment. This requires metagenomic analysis on the basis of sufficient samples to resolve. Second, there is the potential publication bias in this study, although it cannot significantly influence the conclusions. We attribute this limitation to three reasons: ①Incorporating more positive results research, rather than negative/contrary results; ②Sample size; ③Features of follow-up and included population. Third, in retrospective analysis, inherent factors such as patient selection, treatment methods, and drug type/dose affect the heterogeneity of the study. On the basis of sufficient literature, this restriction is expected to be improved through stricter inclusion. Fourth, we did not investigate the correlation between ABX administration and ICI adverse events, which is worth emphasizing in future work. Fifth, due to the study design, impact of other pertinent clinical variables such as age, gender, BMI, PPI use, etc, could not be examined. Finally, in terms of tumor types, our current research mainly focuses on lung cell carcinoma, renal cell carcinoma and melanoma, so we should pay more attention to other solid tumors, such as gastrointestinal or esophageal tumors in the future.

In conclusion, this study evaluated the effect of concomitant ABX use on ICI efficacy in advanced cancer patients by systematically reviewing the relevant literature. The findings demonstrated that ABX use during ICIs treatment of cancer may significantly shorten PFS and OS, and adversely affect the drugs efficacy.

## Data Availability Statement

The original contributions presented in the study are included in the article/supplementary material. Further inquiries can be directed to the corresponding authors.

## Authors Contributions

We declare that all authors made fundamental contributions to the manuscript. All authors contributed to the study conception and design. Database search and data analysis was conducted by SJ, SG, and QC. Study selection and data extraction were performed by CZ, MC, YY, and SZ. The manuscript was written by SJ and SG. NS and MD reviewed the manuscript. All authors read and approved the final manuscript.

## Funding

This work was supported by The Key Program of Harbin Medical University Cancer Hospital Haiyan Fund (no. JJZD2019-03) and The General Program of Harbin Medical University Cancer Hospital Haiyan Fund (no. JJMS2021-25).

## Conflict of Interest

The authors declare that the research was conducted in the absence of any commercial or financial relationships that could be construed as a potential conflict of interest.

## Publisher’s Note

All claims expressed in this article are solely those of the authors and do not necessarily represent those of their affiliated organizations, or those of the publisher, the editors and the reviewers. Any product that may be evaluated in this article, or claim that may be made by its manufacturer, is not guaranteed or endorsed by the publisher.
